# Cross-Regional Comparative Study on Environmental–Economic Efficiency and Driving Forces behind Efficiency Improvement in China: A Multistage Perspective

**DOI:** 10.3390/ijerph16071160

**Published:** 2019-03-31

**Authors:** Xionghe Qin, Yanming Sun

**Affiliations:** 1 Institute for Global Innovation and Development, East China Normal University, 3663 North Zhongshan Rd., Shanghai 200062, China; xionghe@illinois.edu; 2 School of Urban and Regional Science, East China Normal University, Shanghai 200062, China; 3 Institute of Eco-Chongming, East China Normal University, Shanghai 200062, China

**Keywords:** environmental–economic efficiency, efficiency, undesirable output, economic production efficiency, pollution treatment efficiency, network DEA, China

## Abstract

Environmental–economic efficiency assessment is an effective way to evaluate the degree of coordination between an economy and the environment. Previous studies on environmental–economic efficiency have primarily investigated the efficiency of economic production and have often overlooked the efficiency of pollution treatment in overall economic activities. We applied a network data envelopment analysis model to evaluate the environmental–economic efficiency of a multistage process with undesirable outputs in 30 Chinese provinces during 2001–2017. The multistage process consisted of two sequential stages: economic production and pollution treatment. The results show that the average environmental–economic efficiency across all provinces was generally low but demonstrated a gradual upward trend during the study period. The spatial pattern for the 30 provinces showed that provinces with medium or high environmental–economic efficiency are mainly located in the eastern regions in China. Finally, few provinces exhibited economic activities with high economic production and pollution treatment efficiency, with most provinces generally having low economic production and pollution treatment efficiency. Hence, provinces with different economic production and pollution treatment efficiency modes should implement targeted improvement strategies according to their characteristics.

## 1. Introduction

In recent decades, environmental pollution, such as greenhouse gas (GHG) emissions and water wastes, is a major obstacle to sustainable development of society. Meanwhile, the long-standing low efficiency of resource utilization has aggravated the environmental degradation caused by the scarcity of natural resources. The need to effectively address these problems, i.e., to minimize environmental deterioration and use resources more efficiently, has captured the attention of governments and academia worldwide [1].

Since its reform and opening up in 1978, China has achieved remarkable success in both economic and social development, with a gross domestic product (GDP) growth of more than 20-fold over the last four decades. However, this economic development has consumed large amounts of energy and resources, which has led to various environmental issues. For example, China accounted for 25.9% of global greenhouse gas emissions in 2014 [2] (This is based on data for carbon dioxide, methane, nitrous oxide, perfluorocarbon, hydrofluorocarbon, and sulfur hexafluoride emissions compiled by the World Resources Institute). The “Chinese Environmental and Economic Accounting Report 2010” indicates that the economic cost of ecological degradation in China reached 1.54 trillion RMB, accounting for approximately 3.5% of the GDP in 2010. To promote the quality of economic growth, improving energy conservation and emissions reduction as well as increasing energy and environmental efficiency have become major challenges to China’s sustainable development in the future. In this context, policy measures have been implemented by the Chinese government to improve energy utilization and environmental efficiency. Measures adopted involve imposing energy-saving targets and emissions caps set at the inception of the 13th “Five-Year Plan” (2016–2020), adjusting the industrial structure and eliminating excessive capacity of the country. However, relieving environmental stress is complex in light of China’s enormous economic growth, and enhancing economic development while maximally reducing environmental impacts must be considered by Chinese policymakers.

Previous studies have suggested that accurate long-term assessment of economic performance should incorporate both costs resulting from environmental degradation and benefits from environmental improvements [3]. Therefore, indicators that reflect the performance of economic production and pollution treatment should be available to policymakers to allow them to compare the development of countries and regions, set goals, and implement effective policies, both globally and locally [4]. Environmental–economic efficiency evaluation is an efficient way to assess the degree of coordination between the economy and environment. Understanding the coordination between economic production and environmental improvements and their combined overall efficiency aids regional industrial transformation, industrial cooperation, and identification of core problems. This work therefore aims to evaluate regional disparities in environmental–economic efficiency in China, explore their influencing factors, and suggest targeted improvement strategies for regions with different characteristics.

This study contributes to the literature on the assessment of regional environmental–economic efficiency heterogeneity. One frequently employed approach to examine the environmental and economic efficiency is to consider the input and output process of economic activities as a “black box”, focusing on the efficiency of the economic production stage. However, regional economic activities include both the economic production stage and pollution treatment stage. Therefore, analysis of the internal economic activities considering interactions of pollutant treatment processes and their influence on overall efficiency appraisal is an important issue. It allows policy makers to effectively identify inefficiencies in internal processes, which provides basis for policy design.

Using a two-stage network data envelopment analysis model, we evaluated the environmental–economic efficiency of a multistage process for 30 provinces in China during 2001–2017, considering pollutant emissions (undesirable outputs) as critical intermediate outputs. Regional economic activity is a synthesis of economic production and pollution treatment. Based on assessment results, we illustrate the spatial pattern of environmental–economic efficiency for the 30 provinces. We show that provinces with medium or high environmental–economic efficiency are mainly located in eastern regions of China. The decomposition analysis suggests that economic production efficiency in eastern provinces is generally higher than pollution treatment efficiency, whereas provinces with higher values of pollution treatment efficiency are mainly located in northwestern China. We further analyzed factors that have a significant influence on regional environmental–economic efficiency. By highlighting key influencing factors and areas of weakness in economic production and pollution treatment, provinces with different efficiency modes should adjust their improvement strategies correspondingly.

## 2. Literature Review

Environmental–economic efficiency has gained increasing attention from researchers and policymakers. Effectively promoting economic development without harming the environment is a major goal for countries globally. According to the World Business Council for Sustainable Development, the basic concept of environmental–economic efficiency is explained by a comprehensive index measuring environmental impacts accompanied by economic activities. It involves economic products and services as well as environmental resource consumption and emissions. This concept implies that the pursuit of economic growth cannot be solely dependent upon the costs of environmental degradation [5,6]. (Other concepts, such as eco- and energy efficiency, are related to the concept of environmental–economic efficiency. According to the World Business Council for Sustainable Development’s definition, eco-efficiency is achieved through the delivery of “competitively priced goods and services that satisfy human needs and bring quality of life while progressively reducing environmental impacts of goods and resource intensity throughout the entire life-cycle to a level at least in line with the Earth’s estimated carrying capacity”. Critical aspects of eco-efficiency include reduced material and energy intensity of goods or services, reduced dispersion of toxic materials, improved recyclability, maximal use of renewable resources, greater durability of products, and increased service intensity of goods and services. Eco-efficiency has become synonymous with a management philosophy geared towards sustainability, combining ecological and economic efficiency, and is thus wider in its meaning and scope than environmental–economic efficiency. In addition, energy efficiency differs from environmental–economic efficiency in that the primary goal of the former is to reduce the amount of energy required to provide products and services. Improvements in energy efficiency are generally achieved by adopting more efficient technologies and production processes or by applying commonly accepted methods to reduce energy losses. An important motivation to improve energy efficiency is to reduce energy use and promote energy conservation. This may result in a financial cost saving to consumers if the energy savings offset any additional costs of implementing energy-efficient technology.)

The concept of economic efficiency is closely related to the concept of environmental–economic efficiency, as it evaluates the ability of a production unit achieving maximum yield given a set of inputs and production technology. However, in most cases, it may not consider environmental influences or undesirable outputs during production, such as emissions and environmental pollutants, and thus cannot effectively reflect resource consumption and environmental efficiency. In fact, inefficient production activities could result in excessive use of resources and high emissions levels. Hence, environmental–economic efficiency could more comprehensively evaluate the ability to produce more goods and services while reducing natural resource consumption and mitigating environmental impacts [7,8,9].

Assessing environmental–economic efficiency is an effective way to quantitatively judge the performance of economic production and the environment and their interaction. Previous studies have proposed efficiency analysis methods for calculating environmental efficiency, which can mainly be divided into parametric and nonparametric techniques, e.g., stochastic frontier analysis and data envelopment analysis (DEA), respectively. Compared to stochastic frontier analysis, DEA does not need to specify the functional relations between inputs and outputs and is able to measure the relative efficiency of decision-making units (DMUs) with multiple inputs and outputs. It is more easily applied in situations with multiple variables [10,11,12,13,14].

In the literature, different types of DEA models have been employed to evaluate environmental–economic efficiency [15,16,17,18,19]. Based on DEA, total-factor frameworks have been widely used for measuring economy-wide efficiency performance [15]. Generally, the three key input factors, namely capital, labor, and resources, and economic output factors, such as GDP, are included in conventional frameworks [16]. Halkos and Tzeremes [17] used traditional DEA to measure the economic efficiency of the Greek prefectures and revealed regional growth in the 13 administrative regions. Hailu and Veeman [18] argued that traditional measures of economic efficiency only use desirable outputs, such as economic outputs, and ignore undesirable outputs, such as environmental pollutants. As the evaluation of economic efficiency is distorted when human welfare is not considered, most researchers have realized that some of the early estimates of economic efficiency and productivity were biased [19].

Nonetheless, GDP may not be an appropriate metric for measuring the wellbeing of economies [4]. Hence, the study of environmental–economic efficiency, incorporating economic and environmental parameters, may satisfy the need for better measurements or at least provide information about the sustainability of these theories. In response, some researchers have incorporated environmental factors into the traditional DEA model. For example, Hailu and Veeman [20] assumed that environmental pollutant emissions correspond to environmental resources used in production and regarded the emissions of environmental pollutants as special inputs. While this treatment of undesirable outputs may not reflect the real production process, emissions of environmental pollutants as undesirable outputs are expected to be minimized [21,22]. Furthermore, some studies have transformed undesirable outputs into “desirable outputs” [23]. These treatments suffer the problem that the condition where desirable outputs are increased while undesirable outputs are decreased may not be met.

To overcome this defect, Tone [24] developed a more generalized non-radial and non-oriented directional distance function using a slacks-based measure (SBM) considering undesirable outputs. This model has an advantage over the radial and oriented model in previous attempts that either failed to measure potential reductions in undesirable output [25] or could not measure slacks for individual inputs and offer proportionate changes in all inputs to make DMUs efficient [26]. Non-orientation is employed because it can accommodate the simultaneous contraction of inputs and undesirable (bad) links as well as the expansion of outputs.

Furthermore, one limitation of radial models is that radial efficiency does not completely reflect the inefficiency of a DMU [19]. Slacks need to be considered simultaneously with radial efficiency to identify the “real” projection of a DMU. The SBM as a non-radial approach directly accounts for input and output slacks in efficiency measurements, with the advantage of completely capturing the inefficiency. The term “slacks” represents input excesses and output shortfalls and deals with them directly by maximizing these slacks. This property is suitable for analyzing the reduction of undesirable outputs, such as the production of waste gas. In contrast to traditional radial efficiency measures based on the proportional reduction of input (or enlargement of output), the use of SBM (instead of traditional DEA models) allows the analysis to directly deal with input excesses and output shortfalls, called slack measures. The slack measures can capture the non-radial reduction in inputs and non-radial increase in outputs.

Many studies have applied the SBM approach to evaluate environmental–economic efficiency. Wang and Feng [15] employed an undesired-SBM model to analyze the key factors responsible for the change in environmental and economic efficiency in China. Yin et al. [27] considered environmental pollution as an undesirable output and used a super-efficiency model to describe the eco-efficiency of 30 Chinese provincial capital cities. Other studies have dealt only with environmental efficiency and adopted the undesired-SBM model to measure regional environmental efficiency [28,29]. Most studies have focused on the impact of factors such as environmental regulations and production efficiency on environmental efficiency [30,31].

Moreover, previous studies have examined the environmental and economic efficiency problem from different perspectives while considering the input and output process of economic development as a “black box”. However, since actual economic development is not an isolated process and suffers from high environmental costs in many cases, ignoring the undesirable output will not help to identify the real cause of inefficiency [32]. Regional economic activities include both the economic production stage and pollution treatment stage. However, many developing countries and regions generally view the process of economic production as more important than the pollution treatment process. If economic production efficiency is underestimated in these areas, the mitigation of environmental governance—usually accompanied by a loss of economic growth—may not gain much attention from policymakers.

Most published studies have focused on the efficiency of the economic production stage without considering that of the pollution treatment stage. As such, these studies have not considered the internal economic production process, interactions of pollutant treatment processes, or their influence on overall efficiency and were thus unable to effectively identify the efficient stage in economic activities. If the pollution treatment process is ignored, a study concerned solely with either environmental or economic efficiency cannot provide an overall and comprehensive appraisal.

From the viewpoint of a multistage process, environmental–economic efficiency could involve the combination of economic production and pollution treatment efficiency. The former reflects the ability of a production unit to obtain maximum output from a given set of inputs and production technology, whereas the latter represents the degree to which pollutants and emissions from the economic production stage are treated through labor employed, environmental infrastructure investments and other funding.

This study consequently aimed to reconstruct regional economic capacities from a multistage efficiency modeling approach. In contrast to previous studies, this study not only directly examined the relationship between inputs and outputs but also considered linkages of intermediate outputs between different stages. We opened the “black box” of regional economic activity and discuss how a region can achieve superior environmental–economic efficiency. The results could help policymakers understand how economic production and pollution treatment work within a particular economic system and the extent to which the loss of resources and excessive environmental pollution might lead to low environmental–economic efficiency. Regional economic activity is viewed from a multistage perspective and is a synthesis of economic production and pollution treatment. We applied a two-stage network DEA model to a set of 30 Chinese provinces to optimize the overall efficiency of economic production and pollution treatment by considering undesirable outputs (pollutant emissions) as critical intermediate outputs. Therefore, the evaluation framework in this study differs from that in previous research by considering not only the efficiency scores of regional economic production and pollution treatment but also the integrated environmental–economic efficiency in different regions of China. The results could help policymakers understand how economic production and pollution treatment work together within a particular economic system and key factors that contributes to regional environmental–economic efficiency improvement.

## 3. Methods

### 3.1. Conceptual Framework for the Two-Stage Process

**Figure 1:**
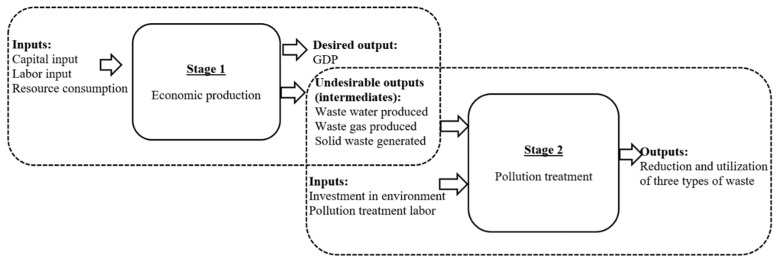
Two-stage process of economic production and pollution treatment with undesirable outputs.

As indicated above, this study sought to develop a two-step analytical procedure for measuring staged environmental–economic efficiency. To accomplish this, a process-oriented conceptual framework of the regional development process needed to be constructed. Economic activity is a complex process and should be evaluated as such, rather than as a single input–output activity. Compared to one-stage models, two-stage models show the performance of individual stages and thus are more informative for decision makers. In this study, following the literature, we divided economic activity into two sub-processes: the economic production process and the pollution treatment process (Figure 1).

Figure 1 illustrates the relationships among inputs, outputs, and intermediate variables. The economic production stage focuses on using resource and non-resource inputs (such as labor and capital) to produce desirable outputs that are accompanied by pollution problems, that is, undesirable outputs (such as wastewater and SO_2_) from the production process in Stage 1. The pollution treatment stage focuses on recycling and disposal of pollution and wastes produced in the first sub-process. There are two types of inputs in the second process. The first involves crucial pollutants that require attention and preferential treatment. These are the intermediate undesirable outputs from Stage 1, which become inputs for Stage 2. The second type is government investment in the form of large amounts of funds and labor provided annually to protect the environment; these investments also enter Stage 1 as an input.

The two sub-processes represent short- and long-term interests, respectively. The economic production stage provides various kinds of daily necessities, such as goods and food, and supports the normal work and life of humankind currently and in the near future. Therefore, this stage represents short-term benefits in the present study. Increasingly, the pollution treatment stage is being established to respond to the requirement of “sustainable development” to perpetually preserve the environment, which represents long-term wellbeing. Environmental–economic efficiency, which is the combination of economic production and pollution treatment efficiencies in this study, can represent both short- and long-term interests.

### 3.2. Network DEA

Previous studies of environmental–economic efficiency considered efficiency evaluation as a “black box” and failed to identify inefficiencies in internal processes. They thus provide little insight into the sources of inefficiencies and the operational stages in which inefficiencies may arise. This study therefore set up a two-stage process cross-region framework for regional economic activities in 30 Chinese provinces. Each province was regarded as a DMU that employs labor, capital, and resources as inputs to produce desirable and undesirable outputs. The environmental–economic efficiency represents the ability of an economic system to translate inputs (labor, capital, and resources) into economic profits and environmental benefits. It is related to the concept of productivity, which improves when the same amount of inputs produces more outputs as a result of efficient resource utilization and allocation.

DEA proposed by Charnes et al. [33] has been used to measure efficiency that considers only the inputs consumed and final outputs produced, ignoring links among sub-DMUs, thus making it difficult to identify ways for DMUs to improve their performance. Therefore, traditional DEA models, such as undesirable-SBM and CCR models [34], apply only to the single-stage efficiency of a system. However, in most real-world situations, DMUs can perform several functions and be separated into different components in series. The entire production process usually includes both economic production and pollution treatment (Figure 1). In this case, some components play important roles in generating outputs by investing intermediate outputs obtained from previous components. Therefore, we proposed a DEA network model that deals with chain relationships among sub-DMUs to measure the environmental–economic efficiency of a two-stage process [35,36].

Previous studies have proposed the network DEA model to use the radial measure of efficiency in the traditional DEA model [37,38]. Tone [24] and Tone and Tsutsui [39] further introduced a non-radial network SBM approach for evaluating efficiency. The advantage of a network SBM is that efficiency decreases strictly monotonically with a change in the degree of input and output slack, and it has the stronger resolution power compared with traditional network DEA [40]. However, intermediate products in this study specifically were undesirable outputs (pollutants) mainly generated by resource inputs in the economic production stage and were treated as inputs in the pollution treatment stage. A two-stage SBM model considering the treatment of undesirable output was applied in this case [41].

It was assumed that there are N DMUs, denoted by DMU_j_ (j = 1, …, N), and each DMU represents an administrative region of China. Furthermore, it was assumed that DMU_j_ has m initial inputs, that is, X = (X_1j_, …, X_mj_)^T^, and g final outputs, that is, H = (H_1j_,…,H_gj_)^T^, with desirable output Y = (Y_1j_, …, Y_sj_)^T^ and intermediate undesirable outputs F = (F_1j_, …, F_dj_)^T^ in the first stage. Undesirable outputs F are used as inputs for the second stage along with a new external input R = (R_1j_, …, R_pj_)^T^. In the economic production stage, policymakers generally hope to obtain more output with less input and discharge less undesirable output. The goal is to achieve the greatest economic efficiency while minimizing undesirable outputs and maximizing desirable outputs.

When evaluating first-stage economic production efficiency based on the SBM model, the intermediate variable *F* as the first-stage undesirable output might be inefficiently slack. Simultaneously, when evaluating second-stage pollution treatment efficiency, the intermediate variable *F* as the second-stage input might also be inefficiently slack, which differs from that in the first stage. That is to say, the slack of *F* in the economic production stage indicates the degree to which the reduction of undesirable output can achieve economic production efficiency, while the slack of *F* in the pollution treatment stage means the degree of excess of pollutant inputs leads to pollution treatment inefficiency. The intermediate variable *F* has different slacks in different stages. Considering this, the slacks in the two stages need to be calculated separately, thereby computing their efficiency.

We defined E_0_ as the environmental–economic efficiency of the two-stage process: $E_{0}^{1}$ is the economic production efficiency in Stage 1, and $E_{0}^{2}$ is the pollution treatment efficiency in Stage 2. The measurement strategy was as follows: First, a network DEA model consisting of two stages was established in Model 1, thereby calculating the slacks of the overall input and output variables. The slack of intermediate variable *F* in Stage 1 (namely undesirable outputs) could be calculated by Model 2 with the unchanged slacks of input and output variables. Consequently, the economic production efficiency in Stage 1 could be calculated using Model 3. Second, the slack of intermediate variable *F* in Stage 2 (namely pollutant inputs) similarly needed to be measured by using Model 4, and Model 5 was subsequently applied to calculate the pollution treatment efficiency in Stage 2. Finally, according to slack variables in Models 1, 3, and 5, Model 6 was used to measure the environmental–economic efficiency in the entire stage.

From the perspective of economic development, pollution treatment forms a crucial part of economic activities. As such, pollution treatment needs to be considered as the second stage of economic activities, thereby acquiring the two-stage efficiency values. If the two-stage process were evaluated as a single process, the condition of the internal sub-processes of economic activities could not be identified, thereby failing to detect the effect of each sub-process on the entire process. Otherwise, when the two sub-processes are separated to evaluate their efficiency individually, the relationship between these sub-processes is not considered. The two-stage efficiency value is calculated based on the measurements of efficiency value in each stage. If the pollution treatment process is ignored, the estimation results of a single stage are not realistic and cause errors. The valuation of two-stage environmental–economic efficiency could enable policymakers to understand how economic production and pollution treatment work within the economic system and the extent to which the loss of resources and excessive environmental pollution might lead to low environmental–economic efficiency.

Accordingly, this study set up a two-stage DEA model based on non-radial SBM to consider undesirable output as follows:

(1) $$E_{0}=\min t-\frac{1}{m+p}(\sum_{i=1}^{m}\frac{S_{i}^{x}}{X_{i0}}+\sum_{i=1}^{p}\frac{S_{i}^{r}}{R_{i0}})\;s.t.\left\{ \begin{array}{l} 1=t+\frac{1}{s+g}(\sum_{i=1}^{s}\frac{S_{i}^{y}}{Y_{i0}}+\sum_{i=1}^{p}\frac{S_{i}^{r}}{R_{i0}})\\ tX_{0}=X\Lambda^{1}+S^{x}\\ tY_{0}=Y\Lambda^{1}-S^{y}\\ tF_{0}\geq F\Lambda^{1}\\ tF_{0}\geq F\Lambda^{2}\\ tR_{0}=R\Lambda^{2}+S^{r}\\ tH_{0}=H\Lambda^{2}-S^{h}\\ S^{x},S^{y},S^{r},S^{h},\Lambda^{1},and\;\Lambda^{2}\geq 0 \end{array}\right.$$

where *s^x^* ≥ 0, *s^y^* ≥ 0, s^f^ ≥ 0, s^r^ ≥ 0, and s^h^ ≥ 0 are slack intensity variables of staged input and output, implying lack of inputs and outputs; and $S_{i}^{x}$ = ${ts}_{I}^{x}$, $S_{i}^{r}$ = ${ts}_{I}^{r}$, $S_{i}^{y}$ = ${ts}_{I}^{y}$, $S_{i}^{h}$ = ${ts}_{I}^{h}$, *Λ*^1^ = t*γ*_1_, *Λ*^2^ = t*γ*_2_, *γ*_1_ and *γ*_2_ are the two-stage intensive vectors.

Under the condition of maintaining constant input and output slack measures in Model 1, the slack measure of undesirable outputs can be obtained as follows:

(2) $$Max\sum_{i=1}^{d}\frac{s_{i}^{f1}}{F_{i0}}\;s.t.\left\{ \begin{array}{l} X_{0}=X\lambda^{1}+s^{x*}\\ Y_{0}=Y\lambda^{1}-s^{y*}\\ F_{0}=F\lambda^{1}+s^{f1}\\ s^{f}\geq 0,\lambda^{1}\geq 0 \end{array}\right.$$

where s^x*^ and s^y*^ are slack variables calculated using Equation (1), and *s^f1^* denotes the slack of the first-stage undesirable output, which represents the degree to which undesirable output can be reduced. In this study, economic production efficiency is defined as follows:

(3) $$E_{0}^{1}=\frac{1-\frac{1}{m}\sum_{i=1}^{m}\frac{s_{i}^{x*}}{X_{i0}}}{1+\frac{1}{s+d}(\sum_{i=1}^{s}\frac{s_{i}^{y*}}{Y_{i0}}+\sum_{i=1}^{d}\frac{s_{i}^{f1*}}{F_{i0}})}$$

If $E_{0}^{1}$ = 1, then DMU_0_ in the first stage is referred to as “efficient”, which indicates that regional economic production efficiency is at its highest. However, Equation (3) only considers the input–output efficiency of the economic production stage and neglects the impact of the internal pollution treatment stage on overall efficiency, which fails to reveal the internal influencing factor of environmental–economic efficiency. Therefore, it is necessary to consider the internal structure of regional economic activity to analyze the overall efficiency of economic production and pollution treatment.

Under the condition of maintaining constant input and output slack measures in Model 2, the slack measure of intermediate products (undesirable outputs from the economic production stage) in the pollution treatment stage can be obtained as follows:

(4) $$Max\sum_{i=1}^{d}\frac{s_{i}^{f2}}{F_{i0}}\;s.t.\left\{ \begin{array}{l} F_{0}=F\lambda^{2}+s^{f2}\\ R_{0}=R\lambda^{2}-s^{r*}\\ H_{0}=H\lambda^{2}-s_{}^{h*}\\ s^{f2}\geq 0,\lambda^{2}\geq 0 \end{array}\right.$$

where s^r*^ and s^h*^ are constant variables calculated using Equation (1), and *s^f2^* denotes the slack variable of the Stage 2 input, namely the undesirable output as intermediate products from the economic production stage. As such, pollution treatment efficiency is defined as follows:

(5) $$E_{0}^{2}=\frac{1-\frac{1}{d+p}(\sum_{i=1}^{d}\frac{s_{i}^{f2*}}{F_{i0}}+\sum_{i=1}^{p}\frac{s_{i}^{r*}}{R_{i0}})}{1+\frac{1}{g}\sum_{i=1}^{g}\frac{s_{i}^{h*}}{H_{i0}}}$$

If $E_{0}^{1}$ = 1, then DMU_0_ in the first stage is referred to as “efficient”, which indicates that regional pollution treatment efficiency is at its highest. However, when economic activity is efficient in each stage of economic production or pollution treatment, this only indicates that inputs, outputs, and intermediate variables in each stage are not slack. Overall environmental–economic efficiency should consider the slack situation of inputs and outputs throughout the entire process as well as that of inputs, outputs, and intermediate products in each stage. Based on the measurements above, overall environmental–economic efficiency can be defined as follows:

(6) $$E_{0}=\frac{1-\frac{1}{m+d+p}(\sum_{i=1}^{m}\frac{s_{i}^{x*}}{X_{i0}}+\sum_{i=1}^{m}\frac{s_{i}^{f1*}}{F_{i0}}+\sum_{i=1}^{m}\frac{s_{i}^{r*}}{R_{i0}})}{1+\frac{1}{s+d+g}(\sum_{i=1}^{s}\frac{s_{i}^{y*}}{Y_{i0}^{}}+\sum_{i=1}^{d}\frac{s_{i}^{f2*}}{F_{i0}}+\sum_{i=1}^{g}\frac{s_{i}^{h*}}{H_{i0}})}$$

We employed the model proposed in this study to measure regional environmental–economic efficiency in each stage separately as well as in the entire process. Compared with a single input–output system, our model could evaluate environmental–economic efficiency in each stage and further provide insights for decision makers. In addition, being different from the two-stage traditional network DEA model proposed by Färe, Grosskopf and Whittaker [36], Tone and Tsutsui [39] and An et al. [42], our model could measure the slack of a two-stage system of input, outputs, and intermediate variables by considering undesirable outputs.

### 3.3. Truncated Regression Model

Since the dependent variable (environmental–economic efficiency) is truncated from below at one, and the independent variables that correspond to 1 can be observed, it has a censored structure. The ordinary least-squares regression model does not provide unbiased and consistent estimates [43]. If OLS regression in the Stage 2 estimation could predict scores greater than one, it might produce biased and inconsistent parameter estimates unless under very peculiar and unusual assumptions about the data-generating process that limit its applicability [44,45]. Put differently, truncated regression with a bootstrapping approach that provides consistent estimation was adopted to examine influencing factors using the following regression model:

(7) $$\hat{\theta }=\alpha +\beta_{i}\;x_{i}+\varepsilon_{i}\;i=1,\;2,\;3,\ldots n$$

where *ε_i_* ~ N (0, $\sigma_{\varepsilon }^{2}$) with left-truncation at 1 − *β_i_ x*_i_; a is a constant term; *x_it_* is the explanatory variable; and *β_i_* is the coefficient of the *i*th influencing factor. For an overall picture and the details of the estimation algorithm, we encourage the interested reader to refer to these studies for details described in a step-by-step approach [44,46,47].

### 3.4. Data Sources and Description

Inputs in the economic production stage are capital, labor, and resources, whereas the output is GDP. Undesirable outputs in the economic production stage are industrial wastes. Capital and labor are traditional input variables used to study economic production efficiency. Since data for capital stock are not available in the China Statistical Yearbook, this study employed a calculation based on the estimation approach suggested by Jun et al. [48]. Estimation results were converted into constant prices with 2000 as the base year. The actual formula used for capital stock in this study was $K_{j}^{t}$ = $I_{j}^{t}$ + (1-δ)$K_{1\;j}^{-t}$, where $K_{j}^{t}$ and $K_{1\;j}^{-t}$ represent the capital stock of the *j*th province at times t and *t*-1, respectively; $I_{j}^{t}$ is the total volume of investment in fixed assets at time t; and δ is the depreciation rate. The China Statistical Yearbook (2002–2018) provides the final number of employees at the end of a particular year. Considering data availability, the total number of employees was used as a proxy for labor in this study.

The study focused on the consumption of resources by regional economic activities, which resulted in more comprehensive input indicators. There are three indicators of resources consumption: area of land used for urban construction, energy consumption, and water consumption. Energy is an important pillar in Chinese economic growth and must therefore be considered. The energy consumption in the study consists of four parts: coal, crude oil, natural gas, and clean energy (i.e., hydropower, nuclear power, and wind power). According to the different calorific value of these energies, total energy consumption was used as an indicator after being converted to standard coal. Freshwater consumption is not included in the traditional model, although it has a large effect on sustainability [49]. As water depletion and waste are becoming increasingly significant in China, freshwater consumption was included in the inputs. Land is a key input that has seldom been considered in previous studies. However, the actual space used and the pattern of utilization vary greatly between years. Therefore, because of the availability of data, this study adopted the area of land used for urban construction as the proxy for urban industrial land use.

The provincial GDP was chosen as “good” output with the data at constant prices (base year = 2000). This served as the indicator of output in the economic production stage. Pollutant production was chosen as the undesirable output. There are various types of pollutants with differences and similarities. Based on Chinese data availability, we chose three types of indicators for pollution: industrial wastewater produced, industrial waste gas produced, and industrial solid waste generated. Since the environmental impact differs from pollutant to pollutant, it provides these pollutants with different importance in the pollution evaluation. In addition, as the units of pollutants are different, it is hard to directly compare the pollution degree caused by these wastes. A potential method to solve this problem is to compare the monetary value of pollutants. However, due to the limitation of data acquisition, it was not possible to assess the monetary value of pollutants from existing official data sources, and only the amount of emissions from different types of pollutants could be obtained. Therefore, to present the different importance of pollutants, this study endowed different pollutants with weights through combination weighting approach of subjective and objective evaluation method (Table 1), and produced the non-dimensional treatment for these indicators, thereby calculating the comprehensive evaluation score of pollutions [50].

The comprehensive evaluation score of pollutions was calculated by the following equation:

(8) $$I_{m}=\sum_{i=1}^{n}w_{i}z_{ij}$$

In Equation (8), *I_m_* represents comprehensive evaluation score, *n* represents the number of indicators for the corresponding criterions, *z_ij_* is the standardized score of each indicator, and *w_i_* is the integrated weight of each indicator.

Stage 2 is the pollution treatment stage, which deals with pollutants when additional contaminant investment is needed to deal with the first-stage undesirable output. Inputs in the pollution treatment stage include two parts: (1) external inputs, that is, pollution treatment labor and investment calculated based on the funding used for environmental infrastructure construction and other fixed assets investment; and (2) internal inputs, which are the undesirable outputs from the economic production stage. Desirable outputs primarily include comprehensive waste removal and utilization, which correspond to three types of expected treated pollution outputs. We used the removal of chemical oxygen demand (COD) and ammonia nitrogen (AN) from industrial wastewater to represent the expected output of industrial wastewater treatment [41]. SO_2_, soot and dust are the main pollutants in industrial waste gas. We used the volume of removed industrial SO_2_, soot and dust as a comprehensive indicator to represent the expected output of industrial waste-gas treatment. The volume of utilized industrial solid waste was selected as the representative desirable output for industrial solid waste treatment in Stage 2. To reflect the difference in the importance of pollutant treatments, the subjective and objective comprehensive weighting method was also used to assess the comprehensive evaluation score of their pollutant treatments (Table 1).

In our study, 30 provinces, municipalities, and autonomous regions were examined. Taiwan, Hong Kong, Macao, and Tibet were excluded because of a lack of data for related indicators. To set targets and develop suitable regional economic and environmental policies, we divided these provinces into three traditional regions (eastern, central, and western regions) according to their economic development and geographic features. The eastern region (11 provinces) is made up of Liaoning, Hebei, Tianjin, Beijing, Shandong, Jiangsu, Shanghai, Zhejiang, Fujian, Guangdong, and Hainan; the central region comprises Shanxi, Jilin, Heilongjiang, Anhui, Jiangxi, Henan, Hubei, and Hunan; and the western region includes Inner Mongolia, Guangxi, Shaanxi, Gansu, Ningxia, Qinghai, Xinjiang, Sichuan, Chongqing, Yunnan, and Guizhou. Environmental–economic efficiency of these regions can be compared to show the regional differences between developed and developing provinces in China.

Unlike in some other countries, Chinese statistical data only become available after a one-year delay. Therefore, the observation period in this analysis was from 2001 to 2017. To comprehensively and accurately measure environmental–economic efficiency, all the input, intermediate, and output variables were considered based on the existing available data. The selection of variables and data sources are shown in Figure 1 and Table 2.

## 4. Results and Discussion

### 4.1. Analysis of Environmental–Economic Efficiency

**Figure 2:**
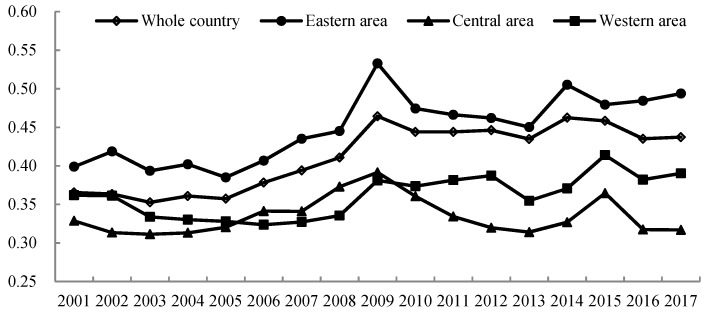
Changing trends of overall environmental–economic efficiency in the eastern, central, and western regions of China, and the whole country during 2001–2017.

Considering undesirable output, we employed a network SBM model to calculate economic production efficiency (ECO_EFCY), pollution treatment efficiency (POL_EFCY), and overall environmental–economic efficiency (E_EFCY) in 30 Chinese provinces from 2001 to 2017. Generally, the average environmental–economic efficiency in the country as a whole over the study period was 0.386 (Figure 2), which was relatively low compared with Shi, Jun, and Wang’s and Zhou et al.’s findings [29,51]. The results reflect the severity of the current ecological problem in China; in other words, the rapid economic growth of China over the study period entailed costs of high resource consumption and environmental destruction [15].

Figure 2 shows the changing trends of overall E_EFCY in the eastern, central, and western regions and the entire country from 2001 to 2017. Overall, E_EFCY showed a gradual upward trend, increasing by 4.2%, from 0.367 in 2001 to 0.409 in 2017. This revealed a significant improvement in the overall environmental–economic efficiency in recent years. Only the eastern region’s average E_EFCY was greater than the national average. The E_EFCY of the eastern region increased considerably over the study period, with an average increase of 9.49%, compared to increase of 2.84% and decrease of 1.18% in the western and central regions, respectively. This suggests that the increase in the overall environmental–economic efficiency of the country as a whole was mainly attributable to the improvement in efficiency in the eastern region. Two factors may contribute to this result: First, it is influenced by the imbalance of economic development, as the eastern region is more developed than the central and western regions. Second, as the eastern region’s industrial structure gradually matures, industries from the eastern region that mostly comprise of high-pollution and -energy consumption sectors are transferred to the western and central regions [30].

### 4.2. Analysis of Two-Stage Efficiency Values

**Figure 3:**
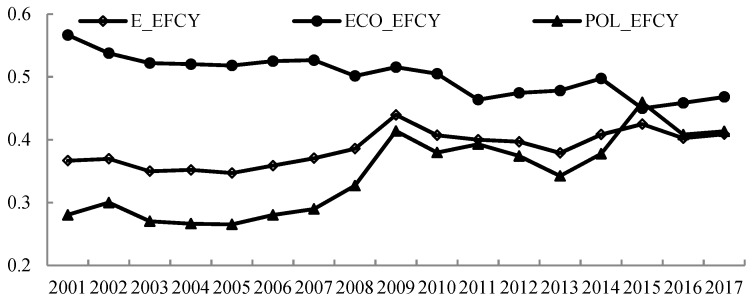
Changing trends of economic production efficiency (ECO_EFCY), pollution treatment efficiency (POL_EFCY), and overall environmental–economic efficiency (E_EFCY) in China from 2001 to 2017.

To reveal the significant differences between the two stages, we present the trends in economic production efficiency and pollution treatment efficiency in time series (Figure 3). As for the decomposition of overall environmental–economic efficiency, the economic production stage had a higher average score and more efficient processes than the pollution treatment stage. It is evident that China overall performed well on the economic front, whereas resource and environmental performance were not encouraging, which is in line with Wang and Feng’s findings [15]. This indicated that economic growth in China has not decoupled from resource inputs and environmental pressure.

Figure 3 shows that ECO_EFCY was at a high level (56.7%) in the base period and decreased annually over the study period. In contrast, POL_EFCY increased noticeably from 0.280 to 0.413 during this period. More specifically, there was slow growth in POL_EFCY before 2006, after which POL_EFCY in mainland China began trending upward. This was attributed to the technological progress over the past few years. With the rapid development of the economy, the Chinese government has gradually increased investment in “high-tech” industries, and a series of preferential policies have been formulated to encourage innovation [52]. An explosion of new technologies and innovations has enabled China to reduce environmental pollutant emissions and enhance pollution control performance. Therefore, during 2001–2017, increased POL_EFCY was the main contributor to the improvement in environmental–economic efficiency.

### 4.3. Spatial Pattern of Regional E_EFCY in China

**Figure 4:**
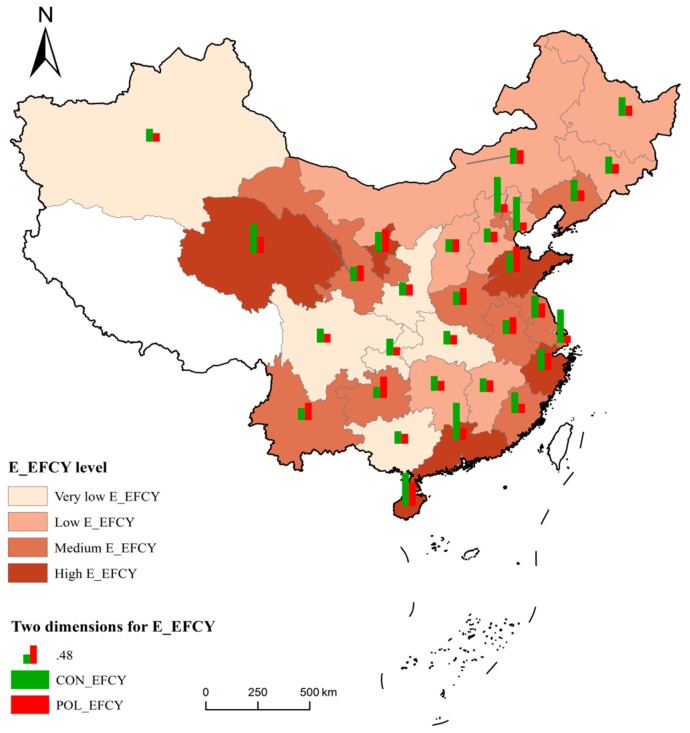
Spatial patterns of environmental–economic efficiency (E_EFCY) in China.

Based on its average score, E_EFCY in the 30 provinces was divided into four levels (high, medium, low, and very low) using the natural breaks classification method (Figure 4) [53]. As shown in Figure 4, provinces with low or very low E_EFCY are mainly located in the northwestern and central regions of China, whereas provinces with medium or high E_EFCY performance are mainly located in the eastern region. Furthermore, mainly in the eastern provinces, the scores of ECO_EFCY were higher than those of POL_EFCY. In contrast, provinces with higher values of POL_EFCY are located in northwestern China. More specifically, some coastal provinces, such as Beijing, Tianjin, Shanghai, and Guangdong, performed well in ECO_EFCY, with average efficiency values of above 0.9, whereas POL_EFCY scores in these provinces were relatively low, with average scores of less than 0.4. This implied that, with current production technology, the overall POL_EFCY in these regions could be improved by more than 60%. However, the scores of POL_EFCY in Hebei, Shanxi, Henan, Yunnan, and Anhui were greater than 0.6, but low ECO_EFCY scores (less than 0.4) led to a moderate overall E_EFCY in these provinces. The heterogeneity of many aspects, such as clean production technology, industrial structure, and environmental regulation, may have resulted in this phenomenon [54]. The technological and management levels of the eastern region are relatively higher, which plays an essential role in reducing resource consumption and mitigating environmental pollution stemming from production. Moreover, the rapid economic development in the developed areas expands the demand for large investments in high-tech industries, which further promotes technological progress and enhances resource utilization.

### 4.4. Analysis of Influencing Factors

Although the environmental–economic efficiency scores are worth presenting on their own, it is even more informative to reveal some of the key determinants in the differences in E_EFCY. Based on existing literature [31,55,56], fine influencing factors were chosen to analyze E_EFCY and were expressed in the logarithmic form (Table 3). This strategy aimed to desensitize the estimation to outliers and allowed for easy interpretation of the estimated coefficients [57].

The influencing factors of environmental–economic efficiency were analyzed using a truncated regression model (Table 4). The advanced industrial structure had a significant influence on E_EFCY, confirming that industrial structure contributed to their high environmental–economic efficiency, which was enhanced by ongoing economic development and upgrades in industrial structures. In many high-income regions, industrial structure changes reduced waste emissions, with a slight increase in the service sector and a decrease in the manufacturing sector [29]. The degree of opening-up had a negative impact on E_EFCY (*p* < 0.01). One possible reason for this phenomenon is that, based on the “pollution haven” and “pollution halo” hypotheses, foreign direct investment could lead to serious environmental pollution and resource consumption in some developing countries and regions but enhance that of others [58]. Specifically, due to economic globalization in China, many energy- and labor-intensive industries have gradually shifted from western countries to China [59]. Particularly, some local governments in China encourage foreign investment in such industries to boost local economies, resulting in an overall negative impact of foreign direct investment on environmental–economic efficiency.

Environmental regulation in the model had a negative but not statistically significant impact on E_EFCY. This point reminds us that improving the environmental–economic efficiency requires resources to be preserved and pollutant emissions to be reduced in Stage 1 rather than the pollution being harnessed in Stage 2. Environmental pollution regulations referred to in this study involve self-financing from enterprises and government subsidies, allowing governmental investment to benefit from pollution regulations. Pollution might be aggravated in cases where the invested capital is applied to increase processing and manufacturing [60]. The coefficient of innovation ability was positive but low, at the 1% significance level, which was consistent with our expectations. Innovation can bring about technological progress, enhance economic production and pollution treatment efficiency, and improve management costs and unexpected output decreases [29]. Enhancing innovation to solve resource and environmental issues is therefore a feasible path.

### 4.5. Environmental–Economic Efficiency Improvement Strategies

**Figure 5:**
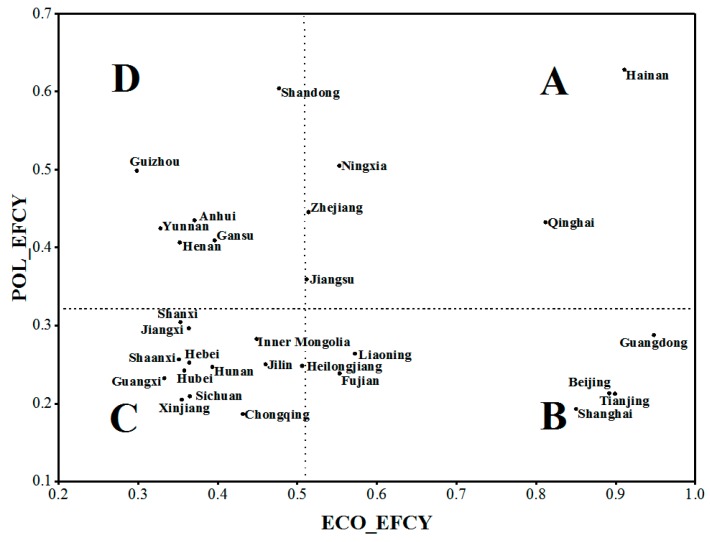
Two-stage environmental–economic efficiency matrix.

The estimated scores of ECO_EFCY and POL_EFCY can be used to identify potential gaps in E_EFCY in the examined provinces and provide specific policy implications for managing regional economic activities. Using the recognized framework of environmental–economic efficiency, which comprises ECO_EFCY and POL_EFCY, we present the most detailed comparative study to date on E_EFCY in Chinese provinces. We plotted two dimensions of E_EFCY to analyze the gap between the dimensions, help discern the locations of the main dimensions influencing E_EFCY and provide improvement strategies at the provincial level (Figure 5). To overcome the problem of selecting arbitrary thresholds for distinguishing high and low ECO_EFCY and POL_EFCY, data were classified based on mean values. The E_EFCY in the 30 provinces were classified into four types: Type A: high ECO_EFCY and high POL_EFCY; Type B: high ECO_EFCY and low POL_EFCY; Type C: low ECO_EFCY and low POL_EFCY; and Type D: low ECO_EFCY and high POL_EFCY.

Overall, thirteen provinces belong to Type C, and five to Type A. Possible optimization strategies based on each type’s efficiency characteristics are described as follows:

- Type A: These provinces have relatively high environmental–economic efficiency in their economic activities and may provide benchmarks of efficiency improvement for other provinces.
- Type B: There are six provinces with low POL_EFCY but high ECO_EFCY. They should maintain their advantage of high ECO_EFCY, while improving POL_EFCY. This may include strengthening research, developing pollutant management, and promoting technological innovation to reduce pollutant emissions.
- Type C: These provinces have invested significant labor and material resources in economic activities. Unfortunately, the benefits have not been fully realized, because they did not focus on factors such as management of economic activities, resource consumption, and pollutant emissions. If these provinces only invest in raising human and resource inputs without implementing policies to increase outputs and reduce pollution, it will be difficult to improve their environmental–economic efficiency.
- Type D: Six provinces emphasize pollution treatment, with relatively low efficiencies in the economic production stage. These patterns point to the need for increased awareness of economic development, including enhancing economic benefits, controlling investment costs, and optimizing resource allocation.

**Figure 6:**
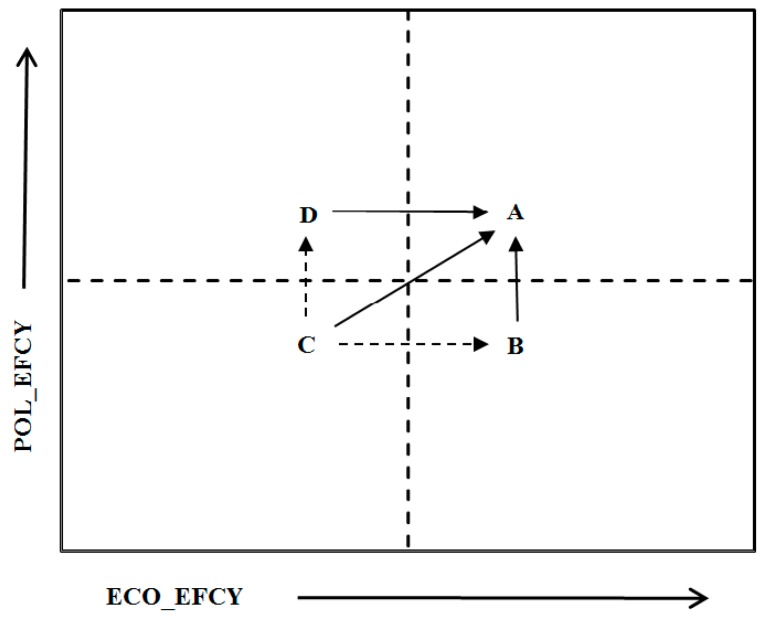
Possible paths for optimization strategy. ECO_EFCY: Economic production efficiency, POL_EFCY: pollution treatment efficiency.

Type B–D provinces have three possible paths for improving efficiencies in such a way that will result in high ECO_EFCY and high POL_EFCY (Figure 6). Path 1 is a unilateral optimization path (B→A; D→A). Type B and C provinces can improve their environmental–economic efficiency by decreasing the disparity with Type A provinces in terms of one weak dimension. Path 2 is a gradually increasing path (C→D→A; C→B→A), where Type C provinces adopt the aforementioned specific policies and flexible measures to first evolve into Types B or D and sequentially progress to Type A. Path 3 is an aggressive optimization path (C→A) that requires provinces to efficiently address weaknesses at each stage of their economic activities simultaneously.

## 5. Conclusions

The purpose of this study was to provide an analysis of regional environmental–economic efficiency from a multistage modeling approach. The “black box” of regional economic activity was opened by considering both the relationship between inputs and outputs as well as intermediate outputs’ linkages between different stages.

In our analysis, regional economic activities involve the economic production process and pollution treatment process. Utilizing a two-stage network DEA model, we measured environmental–economic efficiency at each stage and over the entire process in 30 Chinese provinces from 2001 to 2017. Based on this, we comprehensively analyzed regional environmental–economic efficiency heterogeneity in China, and disclosed particular weaknesses of regions from specific angles, such as economic growth, environmental conservation and pollution control. Potential influencing factors of environmental–economic efficiency were investigated using a truncated regression model. Due to the disparities among provinces, there is no “one size fits all” solution. Our results suggest possible effective paths for region-specific optimization strategy making, which could be based on highlighting areas of weakness in economic production and pollution treatment.

The main conclusions are as follows: First, the average environmental–economic efficiency (E_EFCY) in the country as a whole over the study period was 0.386, and there was a gradual upward trend of the overall E_EFCY. The gap among regions in E_EFCY was significant. The average environmental–economic efficiency of the eastern region was not only greater than the national average but had a larger average increase than that in the central and western regions. This phenomenon in the eastern region is to some extent consistent with the “pollution halo hypothesis”. FDI is a double-edged sword, which transfers seriously polluted industries to developing countries, but also brings advanced technology to these regions. Provinces in the eastern region were more developed than those in the western and central regions. Because production and pollution costs increase in the eastern region, pollution industries may not eventually shift from developed countries through FDI to developed coastal areas in China. FDI may bring advanced environmental protection technology to these areas that focused on resource conservation, production management improvements, and pollution reduction and treatment. However, due to low production and pollution costs, the polluted industries in developed countries are more likely to transfer to the central and western regions through FDI. In addition, China has implemented the industrial transfer policy in recent years, transferring some seriously polluted industries from the developed eastern region to the central and western region, which will reduce the environmental pollution in the eastern region [61].

Second, the decomposition analysis of environmental–economic efficiency shows that China as a whole performed well on the economic front, whereas resource and environmental performances were not encouraging. Since the 10th “Five-Year Plan”, Chinese government has paid much more attention to the improvement of energy utilization and environmental governance. With government’s policy and financial support, the environmental protection industry including waste incineration, waste gas and water treatment has developed strongly. During the study period, growth in pollution treatment efficiency has become a main contributor to the improvement in E_EFCY, whereas the decrease in economic production efficiency is an obstacle. The spatial pattern for the 30 provinces shows that provinces with medium or high E_EFCY performance are mainly located in eastern China. Furthermore, the scores of ECO_EFCY were higher than those of POL_EFCY, mainly in eastern provinces, whereas provinces with higher values of POL_EFCY are located in northwestern China. This showcases the spatial heterogeneity of environmental–economic efficiency. Influencing factor analyses suggest that industrial structure, level of urbanization and innovation ability had a positive influence on environmental–economic efficiency.

Finally, by splitting environmental–economic efficiency into two dimensions, our approach provided a means for understanding and visualizing how each key dimension varied among the 30 provinces, which helped to identify the best practitioners for benchmarking and shed light on ways to improve environmental–economic efficiency by highlighting areas of weakness. Provinces with different efficiency modes could adjust their strategies based on their efficiency characteristics in economic production and pollution treatment accordingly. Additionally, there are three possible paths for provinces without “high ECO_EFCY and high POL_EFCY” characteristics; the best path depends on the regional abilities and current efficiencies.

Our analysis somehow generally supports the Kuznets curve and techno-optimistic theories in the sense that it suggests developed regions have worse technical and environmental–economic efficiency than those less developed ones in China. We especially notice some nuances between our result and the Environmental Kuznets Curve (EKC). First, empirical EKC studies find evidence for an inverted U-shaped pollution-income relation for many pollutants, in particular for short-lived air and water pollutants that have local and immediate effects. In our study, the environmental–economic efficiency concept more comprehensively evaluated the ability to produce more goods and services while reducing natural resource consumption and mitigating environmental pollutions. Second, our study shows that the main reason that developed regions in eastern China have a higher overall environmental–economic efficiency is due to their good performance in the economic production stage. They are actually not better than many central and western provinces in POL_EFCY. For those less developed central and western regions, their relatively lower environmental–economic efficiency is more attributable to lower values of ECO_EFCY, with many of them doing well in the pollution treatment stage.

This study had certain limitations. The inputs of Stage 1 were not applied to Stage 2. Further improvements could consider the inputs of Stage 1 as a shared resource in the inputs of Stage 2. Another limitation, common to comparative efficiency cross-regional studies, is that the observations obtained (i.e., provinces in this study) cannot be completely independent in terms of production input–output correspondence. It must be recognized that there would be spatial spillover effects or spatial correlations in economic efficiency among the 30 provinces. Future research considering these issues would contribute to a better understanding of this phenomenon.

## Figures and Tables

**Table 1:** Subjective weights, objective weights and integrated weights.

Criterion	Indicator	Unit	Type of Weights		
			Subjective Weights	Objective Weights	Integrated Weights
Industrial waste production	Total amount of industrial wastewater produced	10,000 t	0.297	0.302	0.246
	Total amount of industrial waste gas produced	100 million m^3^	0.540	0.331	0.433
	Total amount of industrial solid waste generated	10,000 t	0.163	0.367	0.321
Reduction and utilization of waste	Amount of removal of COD from industrial wastewater	t	0.185	0.208	0.331
	Amount of removal of AN from industrial wastewater	t	0.113	0.239	0.163
	Volume of removed industrial SO_2_	t	0.349	0.217	0.192
	Volume of removed industrial soot and dust	t	0.213	0.163	0.207
	Volume of utilized industrial solid waste	10,000 t	0.140	0.174	0.107

Note: Data from Ministry of Environmental Protection of China. For the calculation of the integrated weight, refer to the work of Qin, Sun, and Zou [50].

**Table 2:** Variable selection and data sources.

Type	Indicator	Variable
Inputs of Stage 1	Capital	Capital stock ^a^
	Labor	Total number of employees ^a^
	Resource consumption	Area of land used for urban construction ^a^
		Energy consumption ^b^
		Water consumption ^b^
Desirable outputs of Stage 1	Economic outputs	GDP ^a^
Undesirable output of Stage 1 (as inputs in Stage 2)	Comprehensive evaluation score of industrial waste production	
Inputs of Stage 2	Investment on environment	Investment used for environmental infrastructure construction and other fixed assets investment ^a^
	Pollution treatment labor	Total number of employees related to environment treatment ^b^
Outputs of Stage 2	Comprehensive evaluation score of reduction and utilization of waste	

Notes: ^a^ Data from National Bureau of Statistics of China. ^b^ Data from. National Energy Statistics Department.

**Table 3:** Influencing factors of environmental–economic efficiency.

Influencing Factors	Index Explanation
1. Industrial structure (*x*_1_)	The proportion of tertiary industry accounts for GDP
2. The degree of opening-up (*x*_2_)	Foreign direct investment
3. Urbanization level (*x*_3_)	The non-agricultural share of total population in every province
4. Environmental regulation (*x*_4_)	The proportion of the investment in environmental pollution regulation to the GDP
5. Innovation ability (*x*_5_)	Number of granted patents

**Table 4:** Results of truncated regression analysis.

Variables	Coefficient	Std. Error	Prob.
*X*_1_	0.0968 *	0.0503	0.054
*X*_2_	−0.0319 ***	0.0065	0.000
*X*_3_	0.2099 ***	0.0804	0.009
*X*_4_	−0.0118	0.0017	0.424
*X*_5_	0.0139 ***	0.0017	0.000

Notes: Level of statistical significance: *** *p* ≤ 0.01, * *p* ≤ 0.1.
